# Evaluation of *Jatropha curcas* L. leaves mulching on wheat growth and biochemical attributes under water stress

**DOI:** 10.1186/s12870-021-03097-0

**Published:** 2021-06-29

**Authors:** Muhammad Irshad, Faizan Ullah, Shah Fahad, Sultan Mehmood, Asif Ullah Khan, Martin Brtnicky, Antonin Kintl, Jiri Holatko, Inam Irshad, Mohamed El-Sharnouby, Ayman EL Sabagh, Rahul Datta, Subhan Danish

**Affiliations:** 1 Department of Botany, University of Science and Technology Bannu, KP Bannu, Pakistan; 2 Hainan Key Laboratory for Sustainable Utilization of Tropical Bio Resource, College of Tropical Crops, Hainan University, Haikou, 570228 China; 3 Department of Agronomy, The University of Haripur, Haripur, 22620 Pakistan; 4 Department of Agrochemistry, Soil Science, Microbiology and Plant Nutrition, Faculty of Agrisciences, Mendel University in Brno, Zemedelska 1, Brno, Czech Republic; 5 Institute of Chemistry and Technology of Environmental Protection, Faculty of Chemistry, Brno University of Technology, Purkynova 118, 612 00 Brno, Czech Republic; 6 Agricultural Research, Ltd., 664 41 Troubsko, Czech Republic; 7 Institute of Soil and Environmental Sciences, University of Agriculture Faisalabad, Faisalabad, 38000 Pakistan; 8 Department of Biotechnology, College of Science, Taif University, P.O. Box 11099, Taif, 21944 Saudi Arabia; 9 Department of Agronomy, Faculty of Agriculture, Kafrelsheikh University, Kafrelsheikh, Egypt; 10 Department of Geology and Pedology, Faculty of Forestry and Wood Technology, Mendel University in Brno, Zemedelska 3, Brno, Czech Republic; 11 Departments of Soil Science, Faculty of Agricultural Sciences and Technology, Bahauddin Zakariya University, Multan, Punjab, 60800 Pakistan

**Keywords:** Mulch, Jatropha, Water stress, Phenolics, Electrolyte leakage, Soluble sugars

## Abstract

**Background:**

Organic mulches are widely used in crop production systems. Due to their benefits in improving soil fertility, retention of soil moisture and weed control. Field experiments were conducted during wheat growing seasons of 2018–2019 and 2019–2020 to evaluate the effects of Jatropha leaves mulch on the growth of wheat varieties ‘Wadan-17’ (rainfed) and ‘Pirsabaq-2013’ (irrigated) under well irrigated and water stress conditions (non-irrigated maintaining 40% soil field capacity). Jatropha mulch was applied to the soil surface at 0, 1, 3 and 5 Mg ha^−1^ before sowing grains in the field. Under conditions of water stress, Jatropha mulch significantly maintained the soil moisture content necessary for normal plant growth.

**Results:**

We noted a decrease in plant height, shoot and root fresh/dry weight, leaf area, leaf relative water content (LRWC), chlorophyll, and carotenoid content due to water stress. However, water stress caused an increase in leaf and root phenolics content, leaf soluble sugars and electrolytes leakage. We observed that Jatropha mulch maintained LRWC, plant height, shoot and root fresh/dry weight, leaf area and chlorophyll content under water stress. Moreover, water stress adverse effects on leaf soluble sugar content and electrolyte leakage were reversed to normal by Jatropha mulch.

**Conclusion:**

Therefore, it may be concluded that Jatropha leaves mulch will minimize water stress adverse effects on wheat by maintaining soil moisture and plant water status.

## Background

Mulch is a coating of organic or inorganic materials applied to soil surface for the conservation of soil moisture, weed control and improving soil fertility [1]. Materials are placed over the soil surface, flower beds, and trees to stop soil erosion on slopes. Mulches are generally applied two inches or deeper [2]. At the start of the growing season, mulches primarily warm the soil, permitting early seeding and transplanting definite crops. The mulch of straw, bark, and sawdust increases soil nutrients [3]. Green mulches, like those prepared from plant parts and animal manure, provide a diversity of nutrients to soil compared to inorganic fertilizers [4, 5]. Moreover, organic mulches help in minimizing fluctuations in soil temperature, providing a microclimate where plants can grow better [6].

Average air temperature on earth has increased over the last 250 years due to a gradually increased industrialization with the concomitant increase in greenhouse gas emissions in the atmosphere. Among scientists, it is extensively accepted that climate change will accelerate in the near future, with expected increases in air temperatures between 1 and 2 °C to occur in the next 40 years [7]. As a result of this, the rate of water loss from the soil surface and plants will be increased [8], which will, in turn, cause a shortage of water supply for agricultural production. Moreover, likely, changes in the rainfall frequencies and patterns will also occur due to the overall climate change. These alterations in rainfall frequencies and patterns will result in scarcity of good quality water and more frequent and extended drought conditions during crop growing [9].

A drought condition can last a few days or can be extended for months or even years [10]. Although an excess of water can also be problematic [11], water scarcity affects far more normal plants' functions, mainly by decreasing their turgor and water potentials [12]. The effects of drought on plants are ubiquitous. Among others, drought in plants results in reduced seed germination [13], the poor seedling establishment [14], decrease in leaf relative water content [15], reduced cell division [16], loss of photosynthetic pigments [17], higher production of secondary metabolites, reduction in the N-fixation rates from the atmosphere [18] and reduced leaf expansion and pollination problems [19, 20]. Cell growth is very sensitive to drought due to a reduction in turgor pressure [21]. In plants, drought stress restricts the uptake of nutrients and their concentration in tissues [22–24], due to reduced transpiration flow and unloading mechanism. Generally, moisture stress causes an increase in nitrogen metabolism, decreasing the phosphorus and potassium content of tissues [25]. Drought stress increases cell wall bound phenolic content which is a reliable sign of drought stress tolerance in plants [26]. Phenolic are secondary metabolites and have several functions in plants. They are structural components of cell walls that enhance tolerance of plants to abiotic stresses [27]. However, under water stress, production of secondary metabolites takes place at the expense of primary metabolites resulting in reductions in the plant biomass production [17].

In the current climate change scenario accompanied by an ever-increasing world population, global energy requirements would need to be doubled in the coming decades. Consequently, cultivation of perennial bioenergy crops [24], or the use of crop residues from corn and other crops [28, 29] will be increased. As a result, regions previously devoted to food crops will be replaced by energy-producing plant species. Therefore, there is a dire need to take sustainable and economic measures to explore the possibility of developing energy-producing plants on a large-scale framework [8].

*Jatropha curcas* L. is an angiosperm that belongs to the *Euphorbiaceae* family. It is inhabitant to Mexico and Central America. The plant has been naturalized and is currently cultivated in tropical and subtropical areas of the world. It can reach a height of up to 6 m, and it is a semi-evergreen, poisonous shrub, or small tree. It was recommended for cultivation in deserts because it is resistant to arid conditions [30]. From the seeds of Jatropha, high-quality biodiesel fuel is produced for use in standard diesel engines. The seeds contain 27–40% oil [31]. Leaves of Jatropha can’t be used as fodder for cattle due to toxins like *Phorbol* esters. However, they contain essential nutrients, which upon their decomposition are released into the soil. There is a lack of information regarding the mechanisms involved in releasing nutrients from decomposing green leaves of *J. curcas* and its potential for providing environmental and agronomic services [32]. Therefore, we attempted to utilize *J. curcas* leaves as mulch for improving the growth and physiological performance of wheat varieties under low soil moisture.

## Results

Jatropha leaf contained natural phenolics (15 mg GAE eq./ g dry weight). Both the macro-and micronutrients such as Ca, Mg, K, Fe, Zn and Mn were found in a reasonable amount. The K content was higher than Ca and Mg. Similarly, Fe content was greater than Zn and Mn (Table 1).

**Table 1:** Composition of Jatropha leaf on dry weight basis

Component	Value
Ca	39 µg/g
Mg	100 µg/g
K	190 µg/g
Fe	45 µg/g
Zn	30 µg/g
Mn	25 µg/g
Phenolics	15 mg GAE eq./g

Our results revealed that Jatropha leaf mulch exhibited a positive effect on soil moisture content (Table 2). All the mulch treatments improved the water holding capacity of soil; however, the highest moisture content 17% was recorded for mulch application at 5 Mg ha^−1^. Water stress decreased soil moisture content by 51% than that of well-irrigated and unmulched control. All the treatments of mulch minimized the decrease in soil moisture content due to water stress. However, the most effective mulch dose in the retention of soil moisture content was 5 Mg ha^−1^. Treatment × variety interaction revealed that both the varieties have a statistically similar response to low soil moisture and mulch treatments. Moreover, on an average of all treatments, variety effect showed that soil moisture content was higher in rhizospheric soil of Wadan-17 (11.980%) than Pirsabaq-2013 (11.650%).

**Table 2:** Soil moisture content as influenced by mulch treatments, varieties and their interactions under water stress

Treatments	Soil moisture content (%)		
	**Wadan-17**	**Pirsabaq-2013**	**Mean**
Control (unmulched and irrigated)	14.373 ± 0.69^c^	14.153 ± 0.16^c^	14.263^c^
Mulch 1 Mg ha^−1^ + water	15.703 ± 0.38^b^	15.313 ± 0.18^b^	15.508^b^
Mulch 3 Mg ha^−1^ + water	15.757 ± 0.29^b^	15.417 ± 0.15^b^	15.587^b^
Mulch 5 Mg ha^−1^ + water	16.597 ± 0.24^a^	16.660 ± 0.11^a^	16.628^a^
Water stress (40% soil field capacity)	7.333 ± 0.23^gh^	6.690 ± 0.07^ h^	7.012^f^
Mulch 1 (Mg ha^−1)^ + water stress	8.337 ± 0.15^ef^	7.870 ± 0.10^ fg^	8.103^e^
Mulch 3 (Mg ha^−1^) + water stress	8.667 ± 0.15^de^	8.233 ± 0.15^ef^	8.450^e^
Mulch 5 (Mg ha^−1^) + water stress	9.077 ± 0.06^d^	8.860 ± 0.06^de^	8.968^d^
Mean	11.980^a^	11.650^b^	

Values of means presented in the same column for different treatments followed by similar English letters don’t differ significantly at *p* < 0.05. Data of two years is pooled due to non-significant variations. Least Significant Difference (LSD): Treatments = 0.5104, Varieties = 0.2552, T × V = 0.7218

Data in Table 3 showed the beneficial effect of Jatropha mulch on the leaf area of wheat. Maximum leaf area (40.1 cm^2^) was recorded for plants supplemented with 5 Mg ha^−1^ of mulch compared with irrigated and unmulched control (32.7 cm^2^). We noted a severe decrease (24.4%) in the leaf area due to skipped irrigations. However, the percent decrease in leaf area was higher in Pirsabaq-2013 than in Wadan-17. It is worthy of mentioning that Jatropha mulch minimized the water stress effect on the leaf area. Among the mulch treatments, the treatment having mulch at 5 Mg ha^−1^ was significantly more effective than other mulch treatments.

**Table 3:** Shoot fresh weight and shoot dry weight of wheat as influenced by mulch treatments, varieties and their interactions under water stress

Treatments	Shoot fresh weight (g)			Shoot dry weight (g)		
	**Wadan-17**	**Pirsabaq-2013**	**Mean**	**Wadan-17**	**Pirsabaq-2013**	**Mean**
Control (unmulched and irrigated	7.007 ± 0.43^c−f^	6.737 ± 0.19^d−f^	6.872^c^	2.4367 ± 0.18^c−e^	2.3900 ± 0.27^c−e^	2.4133^ cd^
Mulch 1 Mg ha^−1^ + water	7.107 ± 0.51^c−e^	7.717 ± 0.36^b−d^	7.412^c^	2.5367 ± 0.11^c−e^	2.7800 ± 0.03^ cd^	2.6583^c^
Mulch 3 Mg ha^−1^ + water	8.327 ± 0.40^bc^	10.490 ± 0.02^a^	9.408^ab^	2.7633 ± 0.10^ cd^	3.2000 ± 0.05^bc^	2.9817^bc^
Mulch 5 Mg ha^−1^ + water	8.737 ± 0.34^b^	11.710 ± 0.89^a^	10.223^a^	2.8000 ± 0.16^ cd^	3.9867 ± 0.65^b^	3.3933^ab^
Water stress (40% FC)	5.497 ± 0.64^ fg^	4.890 ± 0.87^ g^	5.193^d^	2.0567 ± 0.18^de^	1.7700 ± 0.28^e^	1.9133^d^
Mulch (1 Mg ha^−1^) + water stress	5.777 ± 0.17^e−g^	6.877 ± 0.81^c−f^	6.327^c^	2.1700 ± 0.14^de^	2.8300 ± 0.39^ cd^	2.5000^ cd^
Mulch (3 Mg ha^−1^) + water stress	6.060 ± 0.44^e−g^	6.997 ± 0.52^c−f^	6.528^c^	2.1967 ± 0.10^de^	3.0900 ± 0.62^c^	2.6433^c^
Mulch (5 Mg ha^−1^) + water stress	6.107 ± 0.39^e−g^	11.380 ± 0.67^a^	8.743^b^	2.3300 ± 0.14^c−e^	4.9900 ± 0.46^a^	3.6600^a^
Mean	6.8271^b^	8.3496^a^		2.4113^b^	3.1296^a^	

Values of means presented in the same column for different treatments followed by similar English letters don’t differ significantly at *p* < 0.05. Data of two years is pooled due to non-significant variations. Least Significant Difference (LSD) values for shoot fresh weight: Treatments = 1.0893, Varieties = 0.5446, T × V = 1.5405: LSD values for shoot dry weight: Treatments = 0.6205, Varieties = 0.3102, T × V = 0.8774

The non-stressed group plants possessed higher shoot fresh weight (27.0% and 32.8%, respectively) in response to mulch application at 3 and 5 Mg ha^−1^, respectively (Table 3). A significant decrease (24.4%) occurred in shoot fresh weight due to water stress. The percent decrease in fresh shoot weight due to water stress was higher in Pirsabaq-2013 (27.4%) than in Wadan-17 (21.6%). However, the shoot fresh weight of plants growing in plots supplied with Jatropha mulch was not significantly affected by water stress. It was found that mulch application at 5 Mg ha^−1^ resulted in a significantly higher shoot fresh weight of wheat plants both under well irrigated and water stress conditions.

The plants in the non-stressed group had higher root fresh weight (68.6 and 74.6%, respectively) in response to the application of mulch at 3 and 5 Mg ha^−1^, respectively, over irrigated and unmulched control (Table 4). A superior value of fresh root weight was recorded for plants treated with mulch at 5 Mg ha^−1^. A significant decrease (49.8%) occurred in fresh root weight due to water stress. The percent decrease in fresh root weight due to water stress was higher in Pirsabaq-2013 (53.3%) than Wadan-17 (46.1%) as compared with their respective irrigated and unmulched control. However, plants growing in plots supplied with Jatropha mulch were not significantly affected by water stress. Mulch application at the 5 Mg ha^−1^ rate resulted in a significantly higher root fresh weight of wheat plants both under well irrigated and skipped irrigated conditions.

**Table 4:** Root fresh weight and root dry weight of wheat as influenced by mulch treatments, varieties and their interactions under water stress

Treatments	Root fresh weight (g)			Root dry weight (g)		
	**Wadan-17**	**Pirsabaq-2013**	**Mean**	**Wadan-17**	**Pirsabaq-2013**	**Mean**
Control (unmulched and irrigated)	0.4267 ± 0.05^ij^	0.4567 ± 0.00^ij^	0.4417^e^	0.2267 ± 0.03^gh^	0.2467 ± 0.03^gh^	0.2367^e^
Mulch 1 Mg ha^−1^ + water	0.8600 ± 0.06^gh^	1.3800 ± 0.06^b−d^	1.1200^c^	0.5667 ± 0.03^ef^	0.9267 ± 0.03^bc^	0.7467^c^
Mulch 3 Mg ha^−1^ + water	1.2867 ± 0.09^ cd^	1.5267 ± 0.04^bc^	1.4067^b^	0.8367 ± 0.10^ cd^	1.0767 ± 0.05^ab^	0.9567^b^
Mulch 5 Mg ha^−1^ + water	1.8967 ± 0.10^a^	1.5867 ± 0.10^b^	1.7417^a^	1.3100 ± 0.06^a^	1.1800 ± 0.02^a^	1.2450^a^
Water stress (40% soil field capacity)	0.2300 ± 0.01^j^	0.2133 ± 0.01^j^	0.2217^f^	0.1600 ± 0.01^ h^	0.1533 ± 0.00^ h^	0.1567^e^
Mulch (1 Mg ha^−1^) + water stress	0.6900 ± 0.01^hi^	0.8967 ± 0.12^f−h^	0.7933^d^	0.4600 ± 0.02^ fg^	0.6567 ± 0.05^d−f^	0.5583^d^
Mulch (3 Mg ha^−1^) + water stress	0.9867 ± 0.03^e−g^	1.1800 ± 0.16^d−f^	1.0833^c^	0.7000 ± 0.02^c−e^	0.8533 ± 0.13^b−d^	0.7767^c^
Mulch (5 Mg ha^−1^) + water stress	1.1700 ± 0.26^d−f^	1.2333 ± 0.12^de^	1.2017^bc^	0.8567 ± 0.22^b−d^	0.9067 ± 0.12^bc^	0.8817^bc^
Mean	0.9433^b^	1.0592^a^		0.6396^b^	0.7500^a^	

Values of means presented in the same column for different treatments followed by similar English letters don’t differ significantly at *p* < 0.05. Data of two years is pooled due to non-significant variations. Least Significant Difference (LSD) values for root fresh weight: Treatments = 0.2051, Varieties = 0.1026, T × V = 0.2901: LSD values for root dry weight: Treatments = 0.1651, Varieties = 0.0826, T × V = 0.2335

Under well-irrigated conditions, there was an increase (81.0%) in root dry weight due to mulch application at 5 Mg ha^−1^ over unmulched control (Table 4). Water stress decreased root dry weight by 33.8% than irrigated and unmulched control. However, plants growing in plots applied with mulch had a better root dry weight. The highest value of root dry weight was obtained from 5 Mg ha^−1^ mulch application.

Under normal conditions value of LRWC was superior (7.09%) for plants grown in soil amended with mulch at 5 Mg ha^−1^ over irrigated and unmulched control (Table 5). The water stress significantly decreased (6.3%) leaf LRWC than irrigated and unmulched control. The percent decrease in LRWC due to water stress was higher in Pirsabaq-2013 than in Wadan-17. The mulch treatments prevented losses in LRWC under the condition of water stress. LRWC value for Mulch (1 Mg ha^−1^) + water stress is statistically different from the ones obtained with 3 and 5 Mg ha^−1^ mulch + water stress. Of the two wheat varieties, Wadan-17 had a higher value of LRWC than Pirsabaq-2013.

**Table 5:** Leaf relative water content and leaf electrolytes leakage of wheat as influenced by mulch treatments, varieties and their interactions under water stress

Treatments	Leaf relative water content (%)			Leaf electrolytes leakage (%)		
	**Wadan-17**	**Pirsabaq-2013**	**Mean**	**Wadan-17**	**Pirsabaq-2013**	**Mean**
Control (unmulched and irrigated)	70.733 ± 0.74^ab^	62.267 ± 1.27^d^	66.500^b^	99.27 ± 2^de^	119.70 ± 2^b^	109.48^b^
Mulch 1 Mg ha^−1^ + water	72.157 ± 1.11^ab^	64.260 ± 1.64^ cd^	68.208^ab^	92.85 ± 3^ef^	112.38 ± 2^c^	102.62^c^
Mulch 3 Mg ha^−1^ + water	72.403 ± 2.83^ab^	66.913 ± 1.42^b−d^	69.658^ab^	91.00 ± 2^f−h^	104.96 ± 2^d^	97.98^ cd^
Mulch 5 Mg ha^−1^ + water	74.117 ± 1.22^a^	69.037 ± 0.08^a−c^	71.577^a^	84.37 ± 3^hi^	91.37 ± 3^ fg^	87.87^f^
Water stress (40% soil field capacity)	68.773 ± 2.88^a−c^	55.827 ± 1.13^e^	62.300^c^	99.59 ± 2^de^	136.84 ± 1^a^	118.21^a^
Mulch (1 Mg ha^−1^) + water stress	70.200 ± 3.01^ab^	62.410 ± 3.88^d^	66.305^bc^	90.00 ± 3^f−h^	102.70 ± 0^d^	96.35^de^
Mulch (3 Mg ha^−1^) + water stress	70.413 ± 0.22^ab^	62.660 ± 0.09^d^	66.537^b^	85.26 ± 3^ g−i^	100.66 ± 1^d^	92.96^e^
Mulch (5 Mg ha^−1^) + water stress	71.337 ± 0.09^ab^	63.553 ± 3.66^ cd^	67.445^b^	81.08 ± 3^i^	90.58 ± 3^f−h^	85.83^f^
Mean	71.267^a^	63.366^b^		90.43^b^	107.40^a^	

Values of means presented in the same column for different treatments followed by similar English letters don’t differ significantly at *p* < 0.05. Data of two years is pooled due to non-significant variations. Least Significant Difference (LSD) values for leaf relative water content: Treatments = 4.0994, Varieties = 2.0497, T × V = 5.7975: LSD values for leaf electrolyte leakage: Treatments = 4.8954, Varieties = 2.4477, T × V = 6.9232

The electrolyte leakage was decreased by various mulch treatments in leaves of wheat varieties in a controlled environment (Table 5). Electrolyte leakage was highly reduced by applying mulch at 5 Mg ha^-1^ under water stress that mulch treatments overcame. The most effective dose of mulch on electrolyte leakage under water stress was 5 Mg ha^−1^. Wheat variety PirSabaq-2013 had a higher leaf electrolyte leakage than Wadan-17.

Average of both varieties showed that under well-irrigated conditions, Jatropha mulch at 5 Mg ha^−1^ (100.33 cm) significantly increased plant height by 7.8% over well irrigated and unmulched control (93.08 cm) (Table 6). Water stress decreased plant height by 5.8% in water stress (40% FC) than well irrigated and unmulched control. However, mulch application at 3 and 5 Mg ha^−1^ significantly reversed the decrease in plant height caused by water stress. Treatment x variety interaction indicated that a percent decrease in plant height due to water stress was higher in sensitive variety Pirssbaq-2013 than tolerant Wadan-17. Both the varieties have a positive response to mulch at 5 Mg ha^−1^. Variety effect revealed that Pirsabaq-2013 had taller plants (18.2%) than Wadan-17.

**Table 6:** Plant height and leaf area of wheat as influenced by mulch treatments, varieties and their interactions under water stress

Treatments	Plant height (cm)			Leaf area (cm^2^)		
	**Wadan-17**	**Pirsabaq-2013**	**Mean**	**Wadan-17**	**Pirsabaq-2013**	**Mean**
Control (unmulched and irrigated	81.50 ± 2.02^gh^	104.67 ± 2.91^bc^	93.08^b^	29.353 ± 0.61^e^	36.080 ± 1.65^bc^	32.717^c^
Mulch 1 Mg ha^−1^ + water	82.50 ± 2.60^gh^	108.00 ± 0.58^ab^	95.25^ab^	31.487 ± 0.75^de^	36.080 ± 1.65^bc^	33.783^bc^
Mulch 3 Mg ha^−1^ + water	85.33 ± 2.40^f−h^	109.33 ± 5.36^ab^	97.33^ab^	32.477 ± 2.77^c−e^	39.500 ± 1.25^b^	35.988^b^
Mulch 5 Mg ha^−1^ + water	87.00 ± 1.73^ fg^	113.67 ± 1.86^a^	100.33^a^	36.027 ± 1.22^bc^	44.100 ± 0.52^a^	40.063^a^
Water stress (40% FC)	79.33 ± 2.33^ h^	96.00 ± 2.31^de^	87.67^c^	23.367 ± 1.02^f^	26.133 ± 0.35^ef^	24.750^e^
Mulch (1 Mg ha^−1^) + Water stress	87.00 ± 0.58^ fg^	98.00 ± 1.15^ cd^	92.50^bc^	30.377 ± 1.70^e^	35.787 ± 1.92^b−d^	33.082^bc^
Mulch (3 Mg ha^−1^) + water stress	88.5 ± 0.29^ fg^	100.00 ± 1.15^ cd^	94.25^b^	31.140 ± 1.18^e^	37.293 ± 2.02^b^	34.217^bc^
Mulch (5 Mg ha^−1^) + water stress	90.33 ± 2.73^ef^	103.33 ± 4.41^bc^	96.83^ab^	32.327 ± 0.74^c−e^	39.977 ± 1.86^ab^	36.152^b^
Mean	85.19^b^	104.13^a^		30.819^b^	36.869^a^	

Values of means presented in the same column for different treatments followed by similar English letters don’t differ significantly at *p* < 0.05. Data of two years is pooled due to non-significant variations. Least Significant Difference (LSD) values for Soil moisture content: Treatments = 5.1269, Varieties = 2.5634, T × V = 7.2505: LSD values for leaf area: Treatments = 2.9956, Varieties = 1.4978, T × V = 4.2365

Shoot dry weight in wheat was significantly affected by mulch treatments through Jatropha mulch at 5 Mg ha^−1^ presented the largest positive effect (28.9%) increase in dry shoot weight on this factor compared with irrigated and unmulched control (Table 5). Water stress decreased shoot dry weight by 20.7% compared to that of irrigated and unmulched control. However, plants growing in plots applied with mulch had a better shoot dry weight; the highest shoot dry weight was obtained from 5 Mg ha^−1^ mulch application after the water stress period.

In non-stress conditions, mulch treatments significantly improved chlorophyll a content of wheat leaves. However, the highest value of chlorophyll *a* (4.43 mg/g FW) was observed in leaves of plants applied with mulch at 5 Mg ha^−1^ (Table 7). Water stress exhibited a significant decrease (16.1%) in chlorophyll a content. All the mulch treatments alleviated the consequences of water stress on chlorophyll a content; however, the most effective mulch treatments under skipped irrigations were 3 and 5 Mg ha^−1^.

**Table 7:** Leaf chlorophyll a and b content of wheat as influenced by mulch treatments, varieties and their interactions under water stress

Treatments	Chlorophyll *a* (mg/g)			Chlorophyll *b* (mg/g)		
	**Wadan-17**	**Pirsabaq-2013**	**Mean**	**Wadan-17**	**Pirsabaq-2013**	**Mean**
Control (unmulched and irrigated)	1.8867 ± 0.10^de^	3.5633 ± 0.17^bc^	2.7250^ cd^	0.8433 ± 0.03^c^	1.0300 ± 0.17^c^	0.9367^c^
Mulch 1 Mg ha^−1^ + water	2.6967 ± 0.15^c−e^	3.5667 ± 0.19^bc^	3.1317^b−d^	0.9667 ± 0.14^c^	1.2133 ± 0.06^c^	1.0900^c^
Mulch 3 Mg ha^−1^ + water	3.0467 ± 0.58^b−d^	5.6167 ± 0.28^a^	4.3317^a^	1.1467 ± 0.11^c^	1.3733 ± 0.11^bc^	1.2600^bc^
Mulch 5 Mg ha^−1^ + water	3.1467 ± 0.13^bc^	5.7133 ± 0.44^a^	4.4300^a^	1.3633 ± 0.04^bc^	1.3933 ± 0.11^bc^	1.3783^a−c^
Water stress (40% soil field capacity)	1.5767 ± 0.22^e^	2.9967 ± 0.45^b−d^	2.2867^d^	0.7133 ± 0.16^c^	0.9633 ± 0.20^c^	0.8383^c^
Mulch (1 Mg ha^−1^) + water stress	3.2333 ± 0.38^bc^	3.6700 ± 0.54^bc^	3.4517^bc^	0.8767 ± 0.13^c^	1.1767 ± 0.10^c^	1.0267^c^
Mulch (3 Mg ha^−1^) + water stress	3.3033 ± 0.36^bc^	3.8733 ± 0.97^bc^	3.5883^a−c^	2.1333 ± 0.48^ab^	1.2467 ± 0.82^c^	1.6900^ab^
Mulch (5 Mg ha^−1^) + water stress	3.7333 ± 0.47^bc^	3.9633 ± 0.44^b^	3.8483^ab^	2.3267 ± 0.23^a^	1.4267 ± 0.20^bc^	1.8767^a^
Mean	2.8279^b^	4.1204^a^		1.2962^a^	1.2279^a^	

Values of means presented in same column for different treatments followed by similar English letters don’t differ significantly at *p* < 0.05. Data of two years is pooled due to non-significant variations. Least Significant Difference (LSD) values for chlorophyll a: Treatments = 0.8682, Varieties = 0.4341, T × V = 1.2278: LSD values for chlorophyll b: Treatments = 0.5517, Varieties = 0.2758, T × V = 0.7802

We observed that water stress did not affect chlorophyll b content than irrigated unmulched control (Table 7). However, in normal irrigation conditions, mulch showed a positive effect on chlorophyll b content. It was worthy of mentioning that mulch treatments at 3 and 5 Mg ha^−1^ improved chlorophyll b content even under conditions of skipped irrigations.

The plants in control-treated with mulch (mulch + water) showed a significant increase in the leaf carotenoid content compared to unmulched and irrigated control. The gradual increase was recorded in leaf carotenoids content with an increase in mulch quantity. Skipped irrigations resulted in a significant decrease (45.8%) in leaf carotenoids content of both the varieties than their respective irrigated and unmulched control (Table 8). However, mulch treatment Mg ha^−1^ significantly alleviated skipped irrigation's effect on leaf carotenoids content.

**Table 8:** Leaf carotenoids and soluble sugars content (mg/g) of wheat as influenced by mulch treatments, varieties and their interactions under water stress

Treatments	Carotenoids content (mg/g)			Sugars content (mg/g)		
	**Wadan-17**	**Pirsabaq-2013**	**Mean**	**Wadan-17**	**Pirsabaq-2013**	**Mean**
Control (unmulched and irrigated)	0.4267 ± 0.02^f^	0.9633 ± 0.06^a−c^	0.6950^d^	334.40 ± 3^d^	135.00 ± 3^j^	234.70^d^
Mulch 1 Mg ha^−1^ + water	0.8200 ± 0.09^c−e^	0.9767 ± 0.01^a−c^	0.8983^bc^	311.40 ± 3^e^	132.00 ± 2^j^	221.70^e^
Mulch 3 Mg ha^−1^ + water	1.0700 ± 0.02^ab^	1.1000 ± 0.05^a^	1.0850^a^	307.60 ± 5^e^	116.00 ± 2^k^	211.80^f^
Mulch 5 Mg ha^−1^ + water	1.0833 ± 0.04^ab^	1.1133 ± 0.22^a^	1.0983^a^	267.60 ± 0^f^	77.20 ± 0^ l^	172.40^ g^
Water stress (40% soil field capacity)	0.4000 ± 0.03^f^	0.3533 ± 0.04^f^	0.3767^e^	477.60 ± 1^a^	206.20 ± 3^g^	341.90^a^
Mulch (1 Mg ha^−1^) + water stress	0.9100 ± 0.03^a−e^	0.6867 ± 0.11^e^	0.7983^ cd^	450.40 ± 3^b^	167.60 ± 3^h^	309.00^b^
Mulch (3 Mg ha^−1^) + water stress	0.9267 ± 0.10^a−d^	0.7067 ± 0.03^de^	0.8167^ cd^	430.67 ± 5^c^	156.80 ± 2^i^	293.73^c^
Mulch (5 Mg ha^−1^) + water stress	1.1100 ± 0.09^a^	0.8700 ± 0.00^b−e^	0.9900^ab^	335.60 ± 3^d^	118.40 ± 3^k^	227.00^e^
Mean	0.8433^a^	0.8463^a^		364.41^a^	138.65^b^	

Values of means presented in same column for different treatments followed by similar English letters don’t differ significantly at *p* < 0.05. Data of two years is pooled due to non-significant variations. Least Significant Difference (LSD) values for carotenoids: Treatments = 0.1619, Varieties = 0.0810, T × V = 0.2290: LSD values for sugar content: Treatments = 5.8896, Varieties = 2.9448, T × V = 8.3292

In a controlled environment, the soluble sugar content was decreased by various mulch treatments in leaves of wheat varieties (Table 8). A maximum decrease (26.5%) in sugar content was recorded by applying mulch at 5 Mg ha^−1^. Upon exposure to water stress, there was increased sugar content of leaves over unmulched and irrigated control. Mulch treatments overcame the increase in sugar content by skipped irrigations. The most effective dose of mulch on sugar content under skipped irrigations was 5 Mg ha^−1^. Finally, the wheat variety Wadan-17 had a higher leaf sugar content than PirSabaq-2013.

The plants in the control group treated with mulch showed a significant increase in leaf and root phenolics content (Table 9). The highest phenolic content was recorded in leaves and roots of plants treated with mulch at 5 Mg ha^−1^. The water stress also increased the content of leaf phenolics which was further augmented by mulch treatments. The most effective dose of mulch was 5 Mg ha^−1^ under conditions of skipped irrigations.

**Table 9:** Leaf and root phenolics of wheat as influenced by mulch treatments, varieties and their interactions under water stress

Treatments	Leaf phenolics (mg GAE/g f.w)			Root phenolics (mg GAE/g f.w)		
	**Wadan-17**	**Pirsabaq-2013**	**Mean**	**Wadan-17**	**Pirsabaq-2013**	**Mean**
Control (unmulched and irrigated)	19.577 ± 1.85^cd^	6.317 ± 1.40^d^	12.947^d^	40.367 ± 1.55^ef^	22.473 ± 0.52^i^	31.420^g^
Mulch 1 Mg ha^−1^ + water	35.630 ± 2.40^ab^	6.790 ± 0.15^d^	21.210^cd^	68.680 ± 0.70^cd^	24.157 ± 3.31^hi^	46.418^ef^
Mulch 3 Mg ha^−1^ + water	39.527 ± 2.95^a^	10.913 ± 2.55^cd^	25.220^a−c^	75.683 ± 0.49^bc^	33.107 ± 0.64^fg^	54.395^cd^
Mulch 5 Mg ha^−1^ + water	44.317 ± 1.46^a^	21.840 ± 3.13^c^	33.078^ab^	77.193 ± 4.90^bc^	44.737 ± 2.86^e^	60.965^b^
Water stress (40% soil field capacity)	37.263 ± 1.34^a^	6.477 ± 0.29^d^	21.870^cd^	61.367 ± 0.85^d^	23.920 ± 1.93^hi^	42.643^f^
Mulch (1 Mg ha^−1^) + water stress	37.687 ± 10.80^a^	6.527 ± 0.28^d^	22.107^ cd^	70.737 ± 3.04^c^	28.947 ± 3.04^g−i^	49.842^de^
Mulch (3 Mg ha^−1^) + water stress	42.210 ± 1.58^a^	6.683 ± 1.31^d^	24.447^bc^	82.950 ± 3.95^ab^	31.790 ± 0.43^f−h^	57.370^bc^
Mulch (5 Mg ha^−1^) + water stress	44.457 ± 13.46^a^	23.440 ± 1.76^bc^	33.948^a^	86.790 ± 6.53^a^	77.050 ± 3.95^bc^	81.920^a^
Mean	37.583^a^	11.123^b^		70.471^a^	35.773^b^	

Values of means presented in same column for different treatments followed by similar English letters don’t differ significantly at *p* < 0.05. Data of two years is pooled due to non-significant variations. Least Significant Difference (LSD) values for leaf phenolics: Treatments = 9.4646, Varieties = 4.7323, TxV = 13.385: LSD values for root phenolics: Treatments = 6.1090, Varieties = 3.0545, TxV = 8.6395

Data given on Table 10 showed that water stress did not significantly influence leaf antioxidants content of Pirsabaq-2013 but significantly enhanced antioxidant of Wadan-17 (Table 10). The mulch treatments at 5 Mg ha^−1^ significantly improved antioxidants content in leaves of Wadan-17 but did not influence that of Pirsabaq-2013.

**Table 10:** Leaf and root antioxidants content of wheat as influenced by mulch treatments, varieties and their interactions under water stress

Treatments	Leaf antioxidants content (%)			Root antioxidants content (%)		
	**Wadan-17**	**Pirsabaq-2013**	**Mean**	**Wadan-17**	**Pirsabaq-2013**	**Mean**
Control (unmulched and irrigated)	52.447 ± 0.45^e^	72.037 ± 1.58^a^	62.242^c^	29.057 ± 1.83^ g^	41.443 ± 4.23^c−e^	35.250^d^
Mulch 1 Mg ha^−1^ + water	53.000 ± 4.79^e^	71.890 ± 2.31^a^	62.445^c^	30.110 ± 0.71^ g^	42.220 ± 2.31^b−e^	36.165^ cd^
Mulch 3 Mg ha^−1^ + water	56.360 ± 2.61^de^	72.107 ± 0.82^a^	64.233^bc^	31.943 ± 0.42^ fg^	42.333 ± 0.68^b−e^	37.138^ cd^
Mulch 5 Mg ha^−1^ + water	56.693 ± 2.69^c−e^	72.187 ± 0.92^a^	64.440^bc^	38.703 ± 2.18^c−e^	47.890 ± 2.18^ab^	43.297^ab^
Water stress (40% soil field capacity)	60.260 ± 2.66^b−d^	72.223 ± 0.40^a^	66.242^a−c^	36.520 ± 0.52^ef^	42.220 ± 0.64^b−e^	39.370^b−d^
Mulch (1 Mg ha^−1^) + water stress	61.517 ± 2.79^b−d^	70.553 ± 0.76^a^	66.035^a−c^	36.780 ± 0.55^d−f^	42.590 ± 0.98^a−d^	39.685^bc^
Mulch (3 Mg ha^−1^) + water stress	63.150 ± 1.00^bc^	72.443 ± 0.57^a^	67.797^ab^	37.110 ± 2.31^C−F^	42.943 ± 4.59^a−c^	40.027^bc^
Mulch (5 Mg ha^−1^) + water stress	63.260 ± 4.33^b^	75.890 ± 0.71^a^	69.575^a^	42.613 ± 0.55^a−d^	48.500 ± 1.51^a^	45.557^a^
Mean	58.336^b^	72.416^a^		35.355^b^	43.768^a^	

Values of means presented in same column for different treatments followed by similar English letters don’t differ significantly at *p* < 0.05. Data of two years is pooled due to non-significant variations. Least Significant Difference (LSD) values for leaf antioxidants: Treatments = 4.6319, Varieties = 2.3160, TxV = 6.5505: LSD values for root antioxidants: Treatments = 4.2038, Varieties = 2.1019, TxV = 5.9450

Unlike leaf antioxidant content, mulch treatments at 5 Mg ha^−1^ significantly improved the wheat varieties’ root antioxidant content (Table 10). Water stress significantly affects the root antioxidant content of Pirsabaq-2013 and the root antioxidant content of Wadan-17. The progressive impact of mulch was recorded on the root antioxidant content of the Wadan-17. Maximum antioxidant content was recorded in roots of plants treated with mulch at 5 Mg ha^−1^ after water stress.

## Discussion

The experiment confirmed that Jatropha leaves were rich in micro and macronutrients. In addition, he was also satisfactory content of soluble phenols. Mulch is prepared from organic materials for application in farmers’ fields because it contains various kinds of nutrients and phytochemicals necessary to grow crop plants [33]. Previous studies showed that Jatropha leaf contains phenolic compounds having beneficial effects on the growth of wheat [33] and maize [1].

In our study, water stress resulted in the reduction of soil moisture content of field soil. However, Jatropha mulch retained a sufficient amount of soil moisture content necessary for wheat crop growth. Various kinds of organic mulches prepared from plants have been reported effective in retaining soil moisture content in low soil moisture availability. Mulch protects soil moisture content by reducing water loss due to evaporation from the soil surface and better-establishing crops [34].

Plant height and weight were decreased by water stress, which was overcome by Jatropha mulch. Dehydration causes loss of cell turgor, resulting in limited mitotic activity of cells that make plant’s dwarf. We found that Jatropha mulch minimized water loss from soil surface providing normal water supply to plants, thus retaining cells’ turgidity preventing losses in cells' mitotic activity. Moreover, mulch improved the supply of all the major nutrients to wheat plants and prevented a decrease in plant height due to water stress. Similar results were reported in field bean (*Phaseolus vulgaris* L.) applied with newspaper mulch by Ossom and Matsenjwa [35].

We observed a decrease in the leaf area of wheat plants due to water stress. The strength of photosynthesis is directly related to the leaf area. In water stress conditions leaf decreases the transpiration and loss of water by reducing its growth rate. Some researchers showed that a smaller area of the leaf under water stress is inhibiting cell volume. Moreover, water stress causes a reduction in the thickness of spongy and palisade mesophyll tissues in wheat leaf [36]. Remarkably, Jatropha mulch minimized the decrease in leaf area caused by water stress. Organic mulches ultimately break down and mix with soil medium and serve as a nutrients source for plants improving plant growing conditions [37]. In our study, the application of Jatropha mulch improved the leaf area of wheat plants due to higher soil moisture retention, resulting in reduced detrimental impacts of water stress compared to treatments where mulch was not applied.

The relative water content determines the condition of water in cells [38]. Our findings showed that water stress decreased the LRWC of wheat varieties which were remediated by mulch. Reduction in LRWC occurs due to less water availability to plants under water stress conditions [15]. The positive effect of organic mulches on LRWC has been reported earlier [39]. Organic mulches have higher organic matter content, which assists in the water holding capacity of soil [40], contributing to retaining the water status of cells in leaves of wheat during water stress.

We noted a decline in chlorophyll a content due to water stress. However, Jatropha mulch ameliorated the adverse effect of water stress on chlorophyll content. The lower chlorophyll content was recorded in wheat under water stress. The decline of photo-assimilates and pores' closeness under water stress is due to turgor pressure loss [40]. Leaf chlorophyll content was significantly decreased by drought stress [41]. Similar results were earlier reported in rice, okra [42] and wheat [43]. In water stress conditions higher chlorophyll content results in a better seed yield [44, 45]. Mulching caused an increase in soil N and K contents, and the presence of such nutrients has been correlated with the higher production of photosynthetic pigments in plants [46].

We observed that the leaf and root phenolics content of wheat plants were increased due to skipped irrigations. The high buildup of phenolics occurs in plants under conditions of low soil moisture content [17]. Production of phenolics occurs at the cost of photosynthates and results in plants' lower biomass production [26]. However, phenolic compounds function as antioxidants, thus lowering the harmful impacts of reactive oxygen species generated due to environmental stresses [47]. The tolerant variety Wadan-17 exhibited higher content of phenolics in root and leaf. This indicates that phenolic compounds play a significant role in the drought resistance potential of plants. Moreover, mulch treatments showed a positive effect on phenolic production, which may be attributed to the fact that Jatropha leaf was a rich natural phenolic source. This also indicated that wheat plants absorbed phenolic compounds present in the Jatropha mulch from the soil solution, which ultimately increased the level of phenolic compounds in their root and leaf.

We noted an increase in leaf electrolyte leakage of wheat varieties due to skipped irrigations, with a higher electrolyte leakage in the sensitive variety Pirsabaq-2013. Water stress causes loss of cell membrane integrity, and therefore the movement of ions inside and outside the cells is used as an indicator of damage to a great range of tissues [48, 49]. Working on maize plants under drought stress, Valentovic et al. [50] found that membrane damage and electrolyte leakage was higher in leaves of sensitive cultivar than tolerant one. Similarly, Quan et al*.* [51] found that electrolyte leakage was higher in drought-treated maize plants than irrigated ones. Plants treated with Jatropha mulch significantly overcome the increase in electrolyte leakage due to skipped irrigations. Decrease EL in strawberry grown under wheat straw or black polythene has been reported by Kirnak et al*.* [52]. Our results indicate that Jatropha leaf mulch plays an essential role in protecting cell membrane damage that results from skipped irrigations, likely due to the rich concentration of nutrient, phenolic and antioxidant agents may have retained more soil moisture than treatments without mulch application.

Leaf soluble sugar content was increased due to skipped irrigations irrespective of the variety. High production of soluble sugars occurs in plants facing drought stress [53]. Mulch treatments exhibited a decrease in soluble sugar content of wheat plants which may be attributed to the fact that mulch reduced the intensity of water stress caused by skipped irrigations.

## Conclusions

Jatropha leaf was a rich source of natural phenolics and nutrients like Ca, Mg, K, Fe, Zn and Mn necessary for plant growth. Water stress created due to skipped irrigations negatively affected the growth of wheat varieties; however, the severity of water stress was greater on Pirsabaq-2013 than Wadan-17. Plant height, root growth, leaf area and chlorophyll contents increased in mulch-treated soils, particularly those mulched with 5 Mg ha^−1^. Jatropha mulch reversed the increase in leaf electrolyte leakage and soluble sugar content caused due to water stress. Therefore, considering the Jatropha leaf mulching effect on wheat's growth and physiology is an economical choice for future application in the farmer’s field.

## Methods

### Collection of leaves

Fresh leaves of *Jatropha curcas* (20 kg) were collected from 30 mature, healthy plants growing in the Jatropha Garden of the University of Science and Technology Bannu, Pakistan. The leaves were spread on plastic sheets under shade for drying. After drying, the leaves were broken into small pieces and then sieved by using a 2 mm mesh [54].

### Characterization of Jatropha leaves

Jatropha leaves were utilized to investigate the content of total soluble phenolic and mineral nutrients. Total soluble phenolic content was determined by the Folin–Ciocalteau method [55]. A 0.01 g powdered Jatropha leaf was extracted in 5 ml acetone overnight. After centrifugation (3000 × *g*) for 20 min a 130 µl supernatant was added with 130 µl Folin-Ciocalteue reagent, 2.5 ml sodium bicarbonate and 0.5 ml distilled water. After incubation for 90 min, readings of samples were recorded at 765 nm using a spectrophotometer (SP-3 Tokyo, Japan).

For nutrients concentration, a 0.5 g leaf dry leaf powder was digested in 10 ml mixture of nitric acid and perchloric acid (3:1). After digestion of samples, their volume was increased to 50 ml with distilled water. The K, Mg, Zn, Fe, Ca and Mn content was determined by atomic absorption spectroscopy according to Rashid [56] and Ryan et al. [57].

### Seeds sterilization

Certified grains of wheat (*Triticum aestivum* L*.*) varieties Pirsabak-2013 (irrigated) and Wadan-17 (rainfed) were used in this study. Grains were initially sterilized by washing them with 10% solution of Clorox followed by a wash with 95% ethanol for five min.

### Experimental weathering conditions

**Fig. 1:**
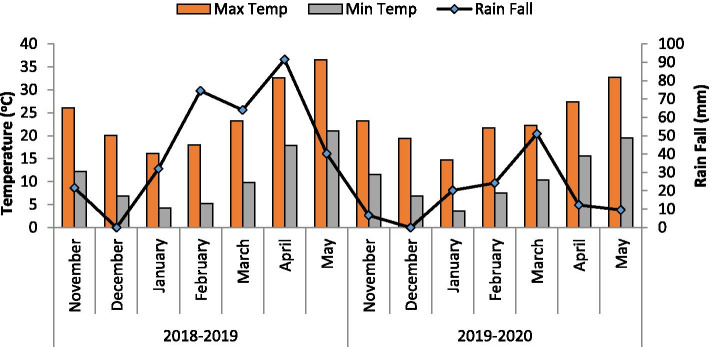
Metrological data of wheat growing seasons of 2018–2019 and 2019–2020

Two field trials were carried out under natural conditions in fields during the wheat growing season of 2018–2019 and 2019–2020 in the prevailing environment of District Bannu, Pakistan. Weather data, including total rainfall and the maximum and minimum air temperatures during the wheat-growing seasons 2018–2019 and 2019–2020, are given in Fig. 1.

Field soil properties were determined by well-established protocols [58–60]. The physicochemical characteristics of soil are given in Table 11.

**Table 11:** Physico-chemical properties of field soil

Parameters	2018–2019	2019–2020
Sand (%)	41	41
Silt (%)	43	42
Clay (%)	15	43
Texture class	Loam	
PH	7.4	7.6
Carbonate	1.8 meq/L	1.9 meq/L
Bicarbonate	2.2 meq/L	2.2 meq/L
Calcium and magnesium	6 meq/L	6.3 meq/L
Chloride	0.8 meq/L	0.7 meq/L
Electric conductivity	250 μS/cm	255 μS/cm
Organic matter	0.052%	0.054%
Nitrogen	0.003%	0.004%

### Design of experiment and treatment plan

The study was carried out as a split-split plot design with three replications. Main plots treatments included a control (irrigated normally when required maintaining 100% soil field capacity) and a water stress treatment, defined as a soil water content equivalent to 40% soil field capacity in this study, resulting from skipping for one and half month around the anthesis stage in wheat; mulch treatments (0, 1, 3 and 5 Mg ha^−1^) were used as the split-plot treatments and two wheat varieties (Wadan-17 and Pirsabaq-2013) as the split-split plots. Plots measuring 1 × 1 m^2^, comprising of four rows each and 5 cm apart, were used. The sowing of grains was made five cm deep in the soil. *Jatropha curcas* leaves' mulch was prepared by making a thin layer on the soil surface one day after sowing wheat grains [61].

### Harvesting and data collection

At the end of the water stress period, wheat characteristics were evaluated at the post-anthesis stage (after flowering). The plant height was determined by using a common measuring tape. Moreover, the root and shoot fresh and dry weight of plants was determined in all treatments. For leaf area measurements, leaf blade length and a leaf blade width of 10 plants per replicate was measured in (cm^2^) separately from every group and replica, and leaf length index was established [62]

Flag leaf was collected from plants randomly in different treatments and analyzed for determination of the leaf relative water content (LRWC) [63], as follows:

FW stands for the flag leaf's fresh weight, DW for dry weight of flag leaf and TW for the turgid weight of flag leaf. The carotenoids and chlorophyll contents of leaves were investigated using the method of Arnon [64] as modified by Kirk and Allen [65]. The pigments were extracted in 80% acetone and analyzed using a spectrophotometer. Leaf and root phenolics content was determined by Folin–Ciocalteau method [66].

The Membrane stability index was determined by the method of Lutts et al. [67]. Leaf discs of 1 cm in diameter were placed in 10 ml deionized water individually. The samples were incubated at room temperature for 24 h on a shaker (100 rpm). After incubation, electrical conductivity (EC1) was determined. A second reading (EC2) was recorded after placing the samples in an autoclave at 120 °C for 20 min and then cooling at room temperature. The following formula determined the electrolyte leakage (EL):

The anti-oxidant potential of roots and leaves was measured by the method of Blois [68]. A 0.1 g leaf or root sample was ground in a 0.5 ml methanol mix with a pestle and mortar. The 5 ml solution was placed overnight in the dark. A 0.1 ml subsample was then taken from the solution, mixed with 2.9 ml DPPH (2,2-diphenyl-1-picrylhydrazyl) solution and placed for 30 min in the dark. Following, antioxidant potentials were taken with a spectrophotometer at 517 nm. Antioxidants (%) was calculated then as follows:

Soluble sugar content in the leaf was determined by using the method of Dubois et al. [69]. A 0.5 g leaf sample was ground with a pestle and mortar. Then, 10 ml of distal water was added and centrifuged at 3000 rpm. Following, a 0.1 ml solution was taken, mixed with 5 ml concentrated H_2_SO_4_, and 1 ml phenol (80%) added to determine soluble sugar content with a spectrophotometer at 420 nm. Calculations were made as follows:

### Statistical analysis

A standard statistical procedure was followed for the comparison of treatments. Two-way ANOVA was used to analyze the data. The mean of three replicates (*n* = 3) comparison was made by the least significant differences test at *p* ≤ 0.05 [70].

## Data Availability

Not applicable.

## Abbreviations

LRWC: Leaf relative water content
f.w.: Fresh weight
N: Nitrogen
Ca: Calcium
Mg: Magnesium
K: Potassium
Fe: Iron
Zn: Zinc
Mg ha^−^^1^: Megagram per hectare

## References

[1] Irshad M, Ullah F, Mehmood S, Khan AU (2016). Jatropha curcas leaves mulch effect on seedling emergence and growth of maize (Zea mays). Sains Malaysiana.

[2] Pittenger D. California master gardener handbook. UCANR Publications; 2014.

[3] Szwedo J, Maszczyk M (2000). Effects of straw-mulching of tree rows on some soil characteristics, mineral nutrient uptake and cropping of sour cherry trees. J Fruit Ornam Plant Res.

[4] Shepherd A, Pickering JS (2000). Evaluation of organic landscape mulches: composition and nutrient release characteristics. Arboric J.

[5] Downer J, Hodel D (2001). The effects of mulching on establishment of syagrus romanzoffiana (Cham.) Becc., Washingtonia robusta H. Wendl. and Archontophoenix cunninghamiana (H. Wendl.) H. Wendl. & Drude in the landscape. Sci Hortic..

[6] andMwangiMaina KA.  (2018). Effects of grass and plastic mulch on growth and yield of strawberries (Fragaria x ananassa) in Kiambu County Kenya. J Anim Plant Sci.

[7] IPCC. Climate change 2007: the physical science basis. Vol 1009. Cambridge University Press: Cambridge; 2007.

[8] Ullah F, Bano A, Nosheen A (2014). Sustainable measures for biodiesel production. Energ Source Part A Recover Util Environ Eff.

[9] Fujihara Y, Tanaka K, Watanabe T, Nagano T, Kojiri T (2008). Assessing the impacts of climate change on the water resources of the Seyhan River Basin in Turkey: use of dynamically downscaled data for hydrologic simulations. J Hydrol.

[10] Jaleel CA, Manivannan P, Sankar B, Kishorekumar A, Gopi R, Somasundaram R (2007). Water deficit stress mitigation by calcium chloride in Catharanthus roseus: effects on oxidative stress, proline metabolism and indole alkaloid accumulation. Colloids Surf B Biointerfaces.

[11] Adeyemi O, Keshavarz-Afshar R, Jahanzad E, Battaglia ML, Luo Y, Sadeghpour A (2020). Effect of wheat cover crop and split nitrogen application on corn yield and nitrogen use efficiency. Agronomy.

[12] Noreen S, Athar HUR, Ashraf M (2013). Interactive effects of watering regimes and exogenously applied osmoprotectants on earliness indices and leaf area index in cotton(Gossypium hirsutum L.) crop. Pakistan J Bot..

[13] Harris D, Pathan AK, Gothkar P, Joshi A, Chivasa W, Nyamudeza P (2001). On-farm seed priming: Using participatory methods to revive and refine a key technology. Agric Syst.

[14] Kaya MD, Okçu G, Atak M, Çikili Y, Kolsarici Ö (2006). Seed treatments to overcome salt and drought stress during germination in sunflower (Helianthus annuus L.). Eur J Agron..

[15] Ullah F, Bano A, Nosheen A (2012). Effects of plant growth regulators on growth and oil quality of canola (Brassica napus L.) under drought stress. Pakistan J Bot..

[16] Avramova V, Abdelgawad H, Zhang Z, Fotschki B, Casadevall R, Vergauwen L (2015). Drought induces distinct growth response, protection, and recovery mechanisms in the maize leaf growth zone. Plant Physiol.

[17] Latif F, Ullah F, Mehmood S, Khattak A, Khan AU, Khan S (2016). Effects of salicylic acid on growth and accumulation of phenolics in Zea mays L. under drought stress. Acta Agric Scand Sect B Soil Plant Sci..

[18] Diatta AA, Thomason WE, Abaye O, Thompson TL, Battaglia ML, Vaughan LJ (2020). Assessment of nitrogen fixation by Mungbean genotypes in different soil textures using 15N natural abundance method. J Soil Sci Plant Nutr..

[19] Battaglia ML, Lee C, Thomason W (2018). Corn yield components and yield responses to defoliation at different row widths. Agron J.

[20] Battaglia M, Lee C, Thomason W, Van Mullekom J (2019). Effects of corn row width and defoliation timing and intensity on canopy light interception. Crop Sci.

[21] Taiz L, Zeiger E (2010). Plant physiology.

[22] Ahanger MA, Morad-Talab N, Abd-Allah EF, Ahmad P, Hajiboland R. Plant growth under drought stress. In: Water stress and crop plants. 2016. p. 649–68.

[23] Adnan M, Fahad S, Zamin M, Shah S, Mian IA, Danish S (2020). Coupling phosphate-solubilizing bacteria with phosphorus supplements improve maize phosphorus acquisition and growth under lime induced salinity stress. Plants.

[24] Diatta AA, Fike JH, Battaglia ML, Galbraith JM, Baig MB (2020). Effects of biochar on soil fertility and crop productivity in arid regions: a review. Arab J Geosci.

[25] Garg BK (2003). Nutrient uptake and management under drought: nutrient-moisture interaction. Curr Agric.

[26] Hura T, Hura K, Ostrowska A, Grzesiak M, Dziurka K (2013). The cell wall-bound phenolics as a biochemical indicator of soil drought resistance in winter triticale. Plant Soil Environ.

[27] Cheynier V, Comte G, Davies KM, Lattanzio V, Martens S (2013). Plant phenolics: recent advances on their biosynthesis, genetics, and ecophysiology. Plant Physiol Biochem.

[28] Battaglia ML, Groover G, Thomason WE. Value and implications of corn stover removal from Virginia fields. In: Virginia Cooperative Extension Publication CSES-180. 2017.

[29] Battaglia M, Groover G, Thomason W. Harvesting and nutrient replacement costs associated with corn stover removal in Virginia. Virginia Tech: Blacksburg; 2018. Available online: https://pubs.ext.vt.edu/content/dam/pubs_ext_vt_edu/CSES/cses-229/CSES-229.pdf.

[30] Gupta MP, Handa SS, Longo G, Rakesh DD. Compendium of medicinal and aromatic plants: The Americas. Unpubl manuscript Gusson, Eduardo. 2003;2:397

[31] Achten WMJ, Verchot L, Franken YJ, Mathijs E, Singh VP, Aerts R (2008). Jatropha bio-diesel production and use. Biomass Bioenerg.

[32] Tidiane D, Komi A, Ibrahima D, Mback eacute S, Amadou LD, Mariama G (2016). The effect of Jatropha curcas L. leaf litter decomposition on soil carbon and nitrogen status and bacterial community structure (Senegal). J Soil Sci Environ Manag..

[33] Khattak A, Ullah F, Wazir SM, Shinwari ZK (2015). Allelopathic potential of Jatropha Curcas l. Leaf aqueous extracts on seedling growth of wheat. Pakistan J Bot..

[34] Kader MA, Senge M, Mojid MA, Ito K (2017). Recent advances in mulching materials and methods for modifying soil environment. Soil Tillage Res.

[35] Ossom E, Matsenjwa V (2007). Influence of mulch on agronomic characteristics, soil properties, disease and insect pest infestation of dry bean (Phaseolus vulgaris L.) in Swaziland. World J Agric Sci..

[36] Han YP, Li YL, Lei ZH, Zhao D, Jia XS (2016). Influence of different root temperature treatment on tomato leaves microstructure. North Hortic.

[37] Gruber S, Acharya D, Claupein W. Wood chips used for weed control in organic farming. In: Journal of Plant Diseases and Proctection, Supplement. 2008. p. 395–400.

[38] Almeselmani M, Abdullah F, Hareri F, Naaesan M, Adel Ammar M, ZuherKanbar O (2011). Effect of drought on different physiological characters and yield component in different varieties of syrian durum wheat. J Agric Sci.

[39] Saeed R, Mirza S, Ahmad R. Electrolyte leakage and relative water content as affected by organic mulch in okra plant (Abelmoschus esculentus (L.) Moench) grown under salinity. 2014. http://fuuastjb.org/index.php/fuuastjb/article/view/173.

[40] Wu FZ, Bao WK, Li FL, Wu N (2008). Effects of water stress and nitrogen supply on leaf gas exchange and fluorescence parameters of Sophora davidii seedlings. Photosynthetica.

[41] Fotovat R, Valizadeh M, Toorchi M (2007). Association between water-use efficiency components and total chlorophyll content (SPAD) in wheat (Triticum aestivum L.) under well-watered and drought stress conditions. J Food Agric Environ..

[42] Amin B, Mahleghah G, Mahmood HMR, Hossein M (2009). Evaluation of interaction effect of drought stress with ascorbate and salicylic acid on some of physiological and biochemical parameters in okra (Hibiscus esculentus L.). Res J Biol Sci..

[43] Moaveni P (2011). Effect of water deficit stress on some physiological traits of wheat (triticum aestivum). Agric Sci Res J..

[44] Alaei Y (2011). The effect of amino acids on leaf chlorophyll content in bread wheat genotypes under drought stress conditions. Middle-East J Sci Res.

[45] Ganji Arjenaki F, Jabbari R, Morshedi A (2012). Evaluation of drought stress on relative water content, chlorophyll content and mineral elements of wheat (Triticum aestivum L.) varieties. Int J Agric Crop Sci..

[46] López R, Burgos P, Hermoso JM, Hormaza JI, González-Fernández JJ (2014). Long term changes in soil properties and enzyme activities after almond shell mulching in avocado organic production. Soil Tillage Res.

[47] Pervez K, Ullah F, Mehmood S, Khattak A (2017). Effect of Moringa oleifera Lam. leaf aqueous extract on growth attributes and cell wall bound phenolics accumulation in maize (Zea mays L.) under drought stress. Kuwait J Sci..

[48] Blokhina O, Virolainen E, Fagerstedt KV (2003). Antioxidants, oxidative damage and oxygen deprivation stress: a review. Ann Bot.

[49] Masoumi A, Kafi M, Khazaei H, Davari K (2010). Effect of drought stress on water status, elecrolyte leakage and enzymatic antioxidants of Kochia (Kochia scoparia) under saline condition. Pakistan J Bot.

[50] Valentovič P, Luxová M, Kolarovič L, Gašparíková O (2006). Effect of osmotic stress on compatible solutes content, membrane stability and water relations in two maize cultivars. Plant Soil Environ.

[51] Cheeseman JM, Lovelock CE (2004). Photosynthetic characteristics of dwarf and fringe Rhizophora mangle L. in a Belizean mangrove. Plant Cell Environ..

[52] Kirnak H, Kaya C, Higgs D, Gercek S (2002). Corrigendum to: A long-term experiment to study the role of mulches in the physiology and macro-nutrition of strawberry grown under water stress. Aust J Agric Res.

[53] Marček T, Hamow KÁ, Végh B, Janda T, Darko E (2019). Metabolic response to drought in six winter wheat genotypes. PLoS ONE..

[54] Weidhuner A, Afshar RK, Luo Y, Battaglia M, Sadeghpour A (2019). Particle size affects nitrogen and carbon estimate of a wheat cover crop. Agron J.

[55] Wolfe K, Wu X, Liu RH (2003). Antioxidant activity of apple peels. J Agric Food Chem.

[56] Rashid A. Mapping zinc fertility of soils using indicator plants and soil analyses. University of Hawaii at Manoa; 1986.

[57] Ryan J, G. Estefan, A. Rashid. Soil and plant analysis laboratory manual. 2nd edn. International Center for Agriculture in Dry Areas (ICARDA) Syria: The National Agricultural Research Center (NARC): Islamabad; 2001. https://www.researchgate.net/publication/236984396_Soil--Plant-AnalysisSoil_and_Plant_Analysis_Laboratory_Manual. Accessed 12 Apr 2020.

[58] Walkley A (1947). A critical examination of a rapid method for determining organic carbon in soils—effect of variations in digestion conditions and of inorganic soil constituents. Soil Sci.

[59] Soltanpour PN, Workman S (1979). Modification of the NH4HCO3-DTPA soil test to omit carbon black1. Commun Soil Sci Plant Anal.

[60] Olsen SR, Sommers LE. Phosphorus. In: Page AL, editor. Method of soil analysis, Agron. No. 9, part 2: chemical and microbiological properties. 2nd edition. Madison: American Society of Agronomy; 1982. p. 403–30.

[61] Kumar R, Sood S, Sharma S, Kasana RC, Pathania VL, Singh B (2014). Effect of plant spacing and organic mulch on growth, yield and quality of natural sweetener plant Stevia and soil fertility in western Himalayas. Int J Plant Prod.

[62] Umar M, Taiwo U (2016). Leaf Area Determinaion for Maize (Zea mays L), Okra (Abelmoschus esculentus L) and Cowpea (Vigna unguiculata L) crops using linear measurements. J Biol Agric Healthc..

[63] Gao JF. Experimental technology in plant physiology. World Map B Press Xi An. 2000;101–3.

[64] Arnon DI (1949). Copper enzymes in isolated chloroplasts Polyphenoloxidase in Beta vulgaris. Plant Physiol.

[65] Kirk JT, Allen RL (1965). Dependence of chloroplast pigment synthesis on protein synthesis: effect of actidione. Biochem Biophys Res Commun.

[66] Singleton VL, Orthofer R L-RR (1999). Analysis of total phenols and other oxidation substrates and antioxidants. Methods Enzym..

[67] Lutts S, Kinet JM, Bouharmont J (1996). NaCl-induced senescence in leaves of rice (Oryza sativaL.) cultivars differing in salinity resistance. Ann Bot..

[68] Blois MS (1958). Antioxidant determinations by the use of a stable free radical [10]. Nature.

[69] Dubois M, Gilles KA, Hamilton JK, Rebers PA, Smith F (1956). Colorimetric method for determination of sugars and related substances. Anal Chem.

[70] Steel RG, Torrie JH, Dickey DA (1997). Principles and procedures of statistics: a biometrical approach.

